# The impact of social support on students' mental health: A new perspective based on fine art majors

**DOI:** 10.3389/fpsyg.2022.994157

**Published:** 2022-11-04

**Authors:** Pengju Wei

**Affiliations:** College of Art and Design, Hainan University, Haikou, China

**Keywords:** college students, mental health, psychological support, social support, social support rating scale

## Abstract

College students face a variety of challenges today, and the degree of their psychological health directly impacts their ability to overcome these challenges. A good psychological state helps college students to invest better in their career development and improve the degree of social integration. This paper uses the SCL-90 Symptom Self-Assessment Scale and the Social Support Rating Scale (SSRS) to investigate the mental health, psychological support, and social support of students from low income backgrounds in two universities in Hainan City. The research results showed that there was no significant difference between the objective support scores of students from low income backgrounds in higher vocational colleges and non-poor students, while the subjective support and utilization of support scores were significantly lower than those of non-poor students. In essence, successful social support should not only be one-dimensional support from the subject to the object, but should be a process of two-sided interaction, or a process of “mutual construction” between supporters and those supported. According to the research conclusions, this paper suggests how to improve the degree of mental health of college students by way of forming a comprehensive educational environment including campus culture construction, ideological and moral education, and economic assistance system.

## Background

### Research background

Adolescent age, which includes school and college-going years, is a crucial period in a student's life when physiological construction, mental change, and social development take place. The level of healthy multi-dimensional development of this age group indirectly and directly impacts peers, families, and communities, which in turn has a butterfly effect on the economy, culture, and wellbeing of the entire society.

Many studies on adolescent age groups typically limit the age of this group to those aged between 7 and 18 years old. However, a growing body of psychological and medical research has found that the development of an individual's brain (frontal lobe) is not fully complete until the age of 25. This finding highlights the upward adjustment of the age range for mental and social relationship health from school-age populations to college student populations. At the university level, students face a whole set of new challenges psychologically. Many college students leave their hometowns and study and live independently in a new place. While these opportunities may positively impact a subset of college students, many studies have found that adapting to living and studying independently for the first time can be a risk factor for depression and anxiety among college students. At the same time, in the 2020 opening semester, the “post-00s” officially entered the university campus as a group of college students. According to data on new students in colleges and universities across China, nearly three-quarters of the first-year undergraduate students in various colleges and universities in 2020 were “post-00s”, which marks a fundamental change in the main body of education in China's colleges and universities. Compared with the “post-80s” and “post-90s”, students from the “post-00s” have more fine material conditions, increasingly diversified value orientations, distinct personality characteristics, a wealthy and prosperous network culture, and other characteristics of the times, signifying a new era and topic for mental health education at colleges and universities.

China's colleges and universities currently attach increasing importance to college students' psychological and behavioral health. After entering the university, the need for academic performance gradually fades and is replaced by whether the person is psychologically healthy, has an independent personality, and socializes. Mental health, behavioral health, and academic performance at the university student level will directly affect this group's career development and social integration. Given the current job scenario, students must establish a healthy mindset, a good personality, a comprehensive and clear understanding of themselves, a suitable career plan, and an accelerated degree of social integration. On the contrary, there can be serious consequences if students are unable to deal with the impact of various adverse events such as getting uprooted and relocated for college education, malignant events on campus, and social pressure, and find it difficult to adapt to the pressure caused by the new environment and emotional fluctuations. Research shows that the proportion of psychological disorders among college students is more than 31%.

Therefore, the need for all-round development of students in terms of mental health, behavioral health, and academic performance is urgent and necessary. Given the large population base of the adolescent age group, and the complex problems facing them, the various development indicators of this demography have increasingly attracted the attention of the academic community. Such concern arises not only because this stage is fundamental to how this age group will develop, but also because it is important to consider how to improve professional intervention methods and facilities on the campus, which have an important impact on youth development. Investing in this age group to ensure their effective development is crucial for the future of China.

### Literature review

A systematic literature review and meta-analysis of multiple studies on psychological and social health interventions at schools generally indicated that evidence-based psychosocial and mental health interventions can effectively alleviate social relationships, mental health, mood, academic level, and many other variables in the student population (Gonzalez-Suarez et al., 2009; Farahmand et al., 2011; Carsley et al., 2018; Sanchez et al., 2018). Based on the available literature, mainstream psychosocial therapy models include cognitive behavioral therapy, problem-solving therapy, motivational interviewing, and short-term focus-resolution therapy. Although other treatment models or genres are also used in student groups, this study primarily focuses on psychotherapeutic models that have more support in the existing empirical literature.

#### Cognitive behavioral therapy

Cognitive behavioral therapy is a psychotherapeutic approach developed in the 1960s by Professor Ellen Baker at the University of Pennsylvania. Cognitive behavioral therapy was initially cognitive therapy. Over time, behavioral therapy and cognitive therapy combined came to be referred to as cognitive behavioral therapy. The cognitive behavioral therapy model mainly involves a process of cognitive reconstruction that helps individuals change “irrational cognition” into “rational cognition.” The cognitive restructuring includes Baker's Cognitive Therapy (CT) and Iris's Rational Behavioral Therapy (REBT). Classical CBT techniques include psychoeducation, cognitive reconstruction, problem-solving, relaxation training, behavioral function analysis, emergency management, exposure, and relapse prevention. According to the type of therapy, it is divided into individual, group, family, and network cognitive behavioral therapy. Baker's cognitive therapy model has made the most significant contributions to the cognitive behavioral therapy model and its many variants and applications. Therefore, existing empirical literature considers Baker to be the founder of cognitive behavioral therapy. Cognitive behavioral therapy has a unique place among many psychotherapeutic genres because cognitive behavioral therapy has the most empirical literature support. As of 2012, the meta-analysis on cognitive behavioral therapy included 269 meta-analyses. These meta-analyses included cognitive behavioral therapy for drug addiction (Riper et al., 2014), depression (Ebert et al., 2015), bipolar disorder, anxiety, somatic disorders (Chiang et al., 2017), eating disorders (Linardon et al., 2017), insomnia (Trauer et al., 2015), personality disorders (Cristea et al., 2017), radical behavior (Battagliese et al., 2015), and many other related disorders (Hofmann et al., 2012).

It is worth mentioning that the effect of cognitive behavioral therapy on various variables of the student population has also been researched extensively. In their study, Ruocco et al. (2016) found that school cognitive behavioral therapy programs significantly improved anxiety symptoms in preschoolers aged between five and seven. Other studies have shown that cognitive behavioral therapy significantly affects behavioral problems and academic improvement in school-age children (Cohen et al., 2018). The effect of cognitive behavioral therapy on students has been widely confirmed by many empirical studies. However, some articles also point caution that cognitive behavioral therapy in student groups also has certain limitations.

#### Problem solving therapy

All the theories and techniques that have a place in the field of psychotherapy are used to solve a client's psychological problems, which is where their vitality lies. In evaluating psychotherapy, a fundamental core question is whether the client's problems can be effectively solved. Because when real-life setbacks and stresses occur, how a person faces and solves these problems has a crucial impact on their physical and mental health. Problem-solving therapy was developed by Nezu and D'Zurilla (2006) based on cognitive behavioral therapy theory and other theories in its genre. Compared with cognitive behavioral therapy, the focus of the problem-solving therapy genre is more short-term. It uses techniques such as coping and behavioral activation of cognitive behavioral therapy to help clients cope with specific stressful events in their lives (Nezu et al., 2012). Problem-solving therapy is a clinical intervention model that improves social coping skills, using a step-by-step guided approach to help clients construct treatments that help them cope with the challenges of daily life (Chibanda et al., 2011). In addition to the behavioral activation component, problem-solving is also concerned with establishing the client's social capacities and social relationships (Wade et al., 2015; Gronholm et al., 2018).

The primary reason for counseling and therapy as a profession is that clients needed help from specialists to help address issues that they are unable to explore on their own. In long-term clinical practice, it has become increasingly clear to experts that psychological counseling and treatment must aim to improve clients' problem-solving skills and independent decision-making. Despite theoretical advancements in counseling and therapy, the minimum goals and outcomes of psychotherapy should be geared toward helping clients effectively solve their problems. Interpersonal Psychology Research shows that the beauty of life is the beauty of interpersonal relationships; the richness of life is the enrichment of interpersonal relationships. Human beings have effective control over their actions; they understand the meaning of self-control, can master skills such as controlling anger and impulse, cultivate a positive mindset, learn stress management and active relaxation, and achieve behavioral self-control. Compared with traditional psychological counseling and treatment systems that require long-term professional training and clinical experience, problem-solving therapy can be summarized as “learning simple, easy, accurate, short, flat, and fast.” The basic process and principles to be followed in complete problem-solving therapy are similar to conventional counseling and treatment. They include six unique sequential problem-solving procedures that help clients resolve their problems (Grant et al., 2001).

#### Motivational interview

Miller and Rollnick (2012) define motivational interview therapy as “a cooperative language that helps enhance a client's motivation and commitment to change.” This treatment model was initially developed by William Miller and Steven Rollick in the development of therapeutic drug addiction. Motivational interviews are based on a respectful and client-centered philosophy of treatment and define, detect, and examine the client's ambivalence in making behavioral changes by employing core clinical techniques (Miller and Rose, 2015). Motivational interview therapy and Carl Rogers' humanistic therapy are very similar and mainly employ four core principles of intervention: 1. express empathy, 2. identify inconsistencies in existing behavior and critical goals, 3. allow clients to express freely, to refuse or be unsure 4. discuss a change plan.

The most significant advantage of MI is that it integrates the advantages of various existing therapeutic theories and techniques and has developed unique techniques of consultation and treatment. Compared with traditional treatment methods for similar health problems, the effect is fast and effective. The degree of consolidation of behavioral change is higher, and it is widely used and generally recognized in the west. However, China has a unique social and cultural background, which involves different ways of addressing the world to other countries. If MI was adopted at present, it would be difficult for it to be effective in China culturally. For example, Zen Buddhism's view of human nature is similar to humanism, but the way the two work is dissimilar as they involve different cultural backgrounds. The question of how best to incorporate such cultural phenomena into systematic and rigorous scientific approaches requires in-depth exploration and research.

Given the above issues, the influence of cultural factors on the intervention process has become significant, and localizing MI requires much effort. At present, research in China on MI is underdeveloped, the scope of application is narrow, and it cannot play an active role in a broader range of fields. Motivational interviews, therefore, require more in-depth research by psychologists and broader clinical practice for application in China.

## Research methods

### Research sample

In this paper, students from two universities in Hainan were selected for investigation. There were about 550 students in 2020 and 2021, aged between 18 and 22, majoring in fine arts, and 530 valid answers were obtained. See Table 1 for sample distribution.

**Table 1:** Sample distribution.

	**Male**	**Female**	**Urban**	**Rural**	**Poverty**	**Non-poverty**
Number of students	295	235	310	220	78	452
Proportion (%)	55.66	44.34	58.49	41.51	14.72	85.28

### Research tools

A questionnaire was distributed to the sample population. It included a section that collected basic information about students, including gender, age, source, urban or rural, and monthly spending. According to the average minimum living standard of residents in the student area, students spend <200 yuan per month. Based on this spending pattern, the college students were divided into low income college students and non-poor college students, to compare and analyze the psychological status of poor and non-poor students. The questionnaire included the following two scales.

#### SCL-90 symptom self-assessment scale

The SCL-90 Symptom Self-Assessment Scale was compiled by Derogatis. L.R. (1975) and is widely used in several mental health condition surveys. The scale has a total of 90 items, testing the content of ten factors of students, and the symptoms (usually occurring at the time of day or week) reflected in this scale are rich and specific. Each item is scored on a scale of one to five, ranging from No (1), very light (2), moderate (3), heavy (4), to severe (5). The ten factors are:

(1) Somatization factor: This factor mainly reflects subjective physical discomfort, including cardiovascular, gastrointestinal, and respiratory system complaints of discomfort, headache, spinal pain, muscle soreness, and other physical manifestations of anxiety.
(2) Obsessive-compulsive symptom: This factor mainly refers to meaningless thoughts, impulsive behaviors, and other manifestations that are not necessary but cannot be shaken off, and some more general perceptual disorders (such as “the brain has become empty,” and “memory is not good,”) are also reflected in this factor.
(3) Interpersonal sensitivity: This factor mainly reflects some individuals' feelings of discomfort and inferiority, especially when compared with others. People with low self-esteem, chagrin, and poor relations with each other are usually characterized by this factor.
(4) Depressive factor: This factor reflects a broad concept of clinical depressive symptom groups. Melancholy and depressed feelings and moods are representative symptoms. It is also characterized by decreased interest in life, a lack of desire to be active, a loss of mobility, etc. It includes disappointment, pessimism, and other sensory and physical problems associated with depression. Several of the items in this factor include concepts such as death and suicide.
(5) Anxiety factor: This factor includes some manifestations and experiences that are usually clinically obviously associated with anxiety symptoms, and generally refers to those who are unable to rest, are nervous, and exhibit somatic signs such as tremors. Manifestations of wandering anxiety and panic attacks are the main elements of this factor.
(6) Hostile factors: This factor mainly reflects the expression of hostile thoughts, feelings, and behaviors, and ranges from boredom, arguments, and throwing objects to fights and irrepressible bursts of impulse.
(7) Terror factor: This factor is reflected through a state of being terrified; the object of fear includes travel, open space, crowds, public places, and means of transportation. Some projects reflect social horror.
(8) Paranoia factor: Paranoia is a very complex concept; this factor only includes some of its primary content, and mainly refers to the aspect of thinking, such as projection thinking, hostility, suspicion, relationship delusion, delusion, passive experience, and praise.
(9) Psychosis: It includes among others, fantasies, the spread of thoughts, a sense of control, and thinking that reflect the selective symptoms of schizophrenia, which are used to quickly and concisely understand the degree of the disease.
(10) Other: This factor reflects issues related to sleep and diet.

#### Social support rating scale

Since 1986, the Social Support Rating Scale has been applied in dozens of studies in China. From its feedback, it is accepted that the questionnaire is well-designed, the entries are easy to understand and unambiguous, and have good reliability and validity. Social support is an essential factor affecting people's social life, and the scale has ten items, including three dimensions: objective support (three items), emotional support (four items), and utilization of social support (three items). The level of social support that individuals expect and receive can be understood through this scale, which can help people to adapt to society and the environment better and improve their physical and mental health. To customize the questionnaire to college students, this paper modified some text entries in the scale; in the fourth question, “colleague” was replaced with “classmate,” “couple” with “lover,” and removed “children,” and “spouse” in questions 6 and 7 were replaced with “lover,” and “parents” were added. After receiving the questionnaires, the statistical analysis was performed using SPSS.

## Statistical results and analysis

### Survey results and analysis of the SCL-90 symptom self-evaluation scale

#### Results of SCL-90 factors for needy and non-poor students

**Figure 1:**
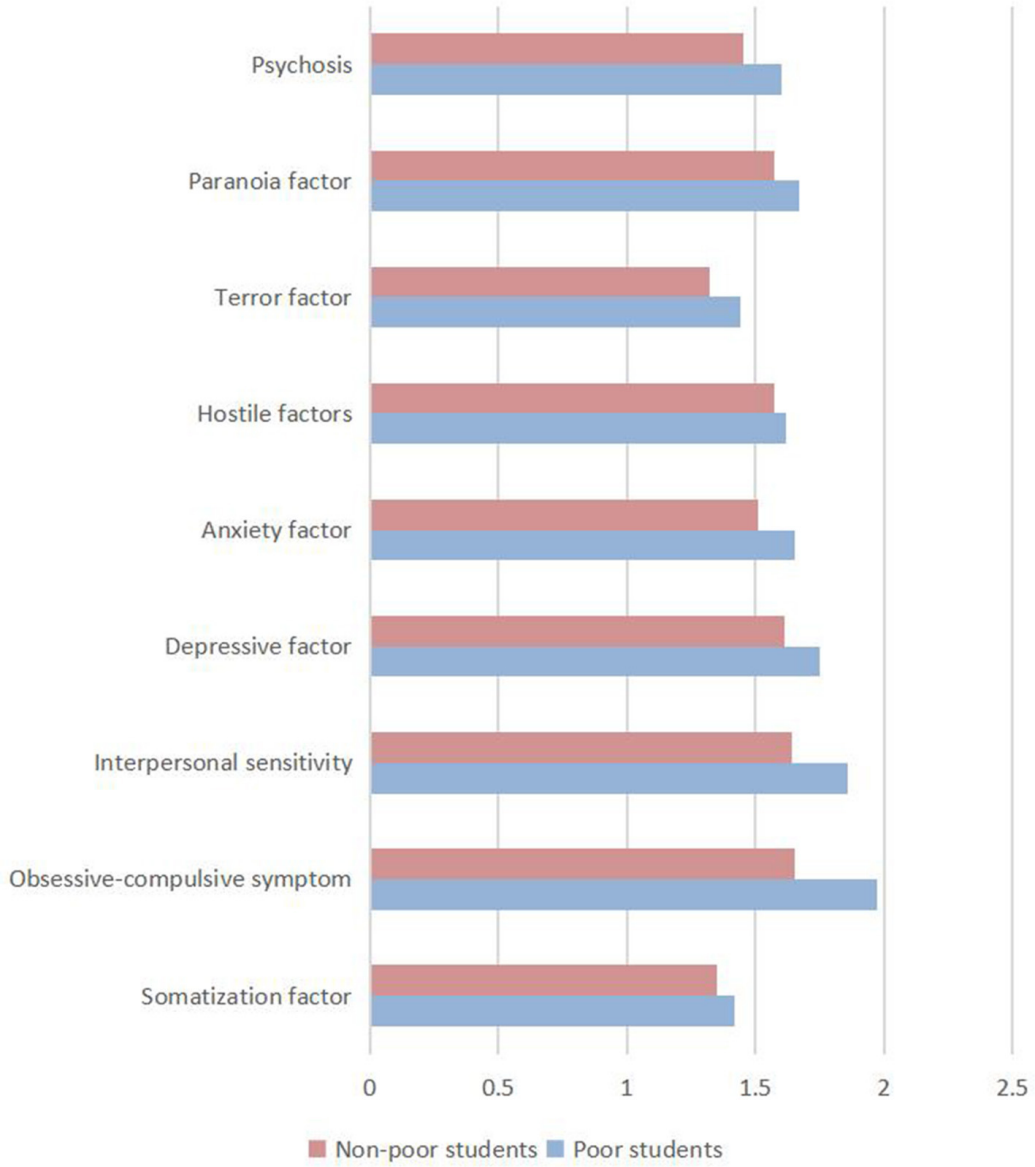
The results of SCL-90 factors for poor and non-poor students.

As seen in Table 2 and Figure 1, there is no significant difference in the terror factors between the students from low income backgrounds and non-poor students in the two universities in Hainan city. However, the average score of the remaining eight factors is significantly higher in poor students.

**Table 2:** Results of SCL-90 factors for poor and non-poor students.

**Factor**	**Needy students (78)**	**Non-poor students (452)**	***t***
Somatization factor	1.42 ± 0.44	1.35 ± 0.41	1.9466*
Obsessive-compulsive symptom	1.97 ± 0.57	1.65 ± 0.52	3.1347**
Interpersonal sensitivity	1.86 ± 0.59	1.64 ± 0.51	4.6453**
Depressive factor	1.75 ± 0.62	1.61 ± 0.49	3.6757**
Anxiety factor	1.65 ± 0.55	1.51 ± 0.49	2.8314**
Hostile factors	1.62 ± 0.52	1.57 ± 0.57	1.2834*
Terror factor	1.44 ± 0.45	1.32 ± 0.45	2.3911
Paranoia factor	1.67 ± 0.50	1.57 ± 0.48	2.6658**
Psychosis	1.60 ± 0.54	1.45 ± 0.43	5.5776**

*Indicates *P* < 0.05, **Indicates *P* < 0.01, and ***Indicates *P* < 0.001. *P* < 0.05, that is, indicates a statistically significant difference. *P* < 0.01 indicates a statistically significant difference. *P* > 0.05 indicates no statistically significant difference.

#### Positive detection rate of SCL-90 factors among needy and non-poor students

**Figure 2:**
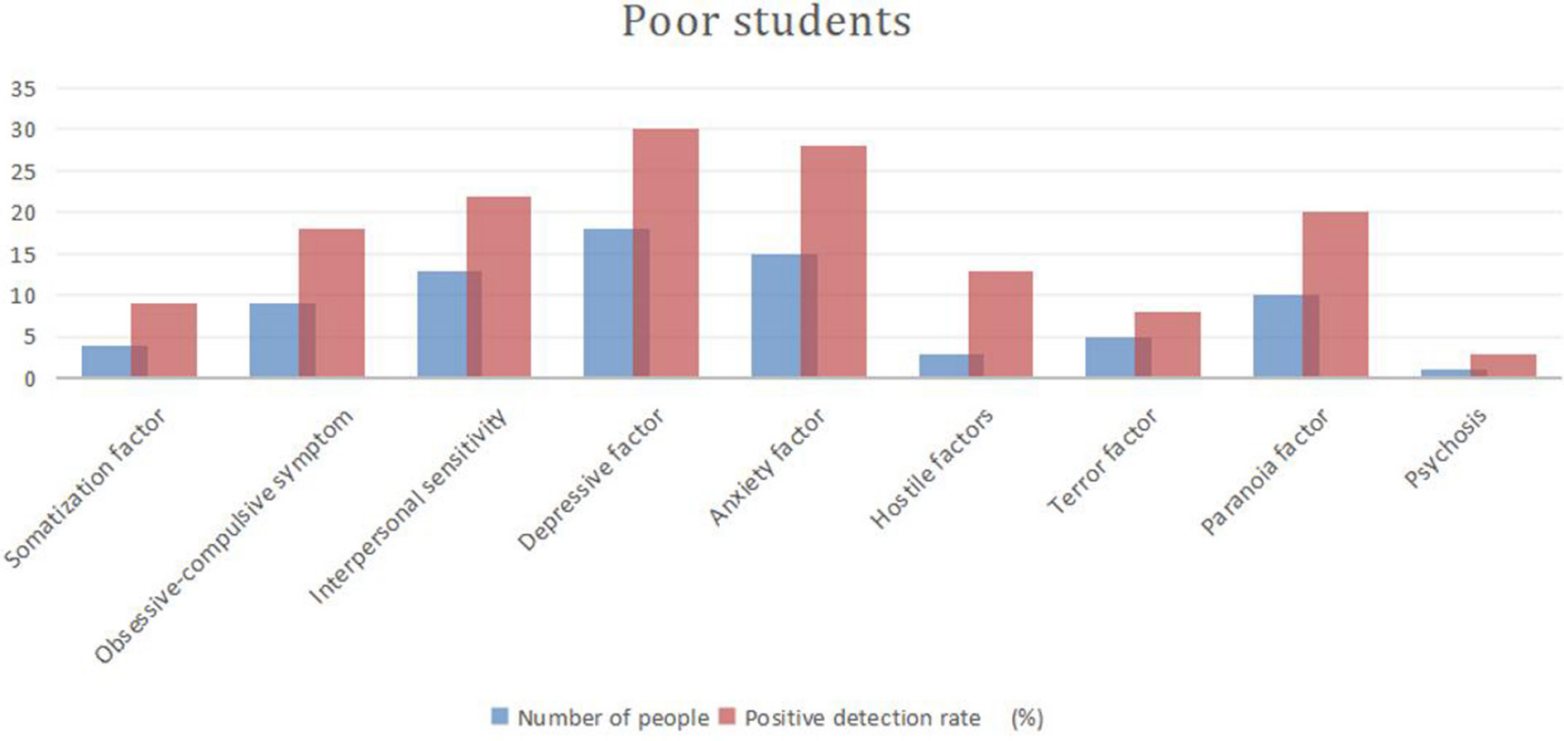
Positive detection rates of SCL-90 factors among needy students.

**Figure 3:**
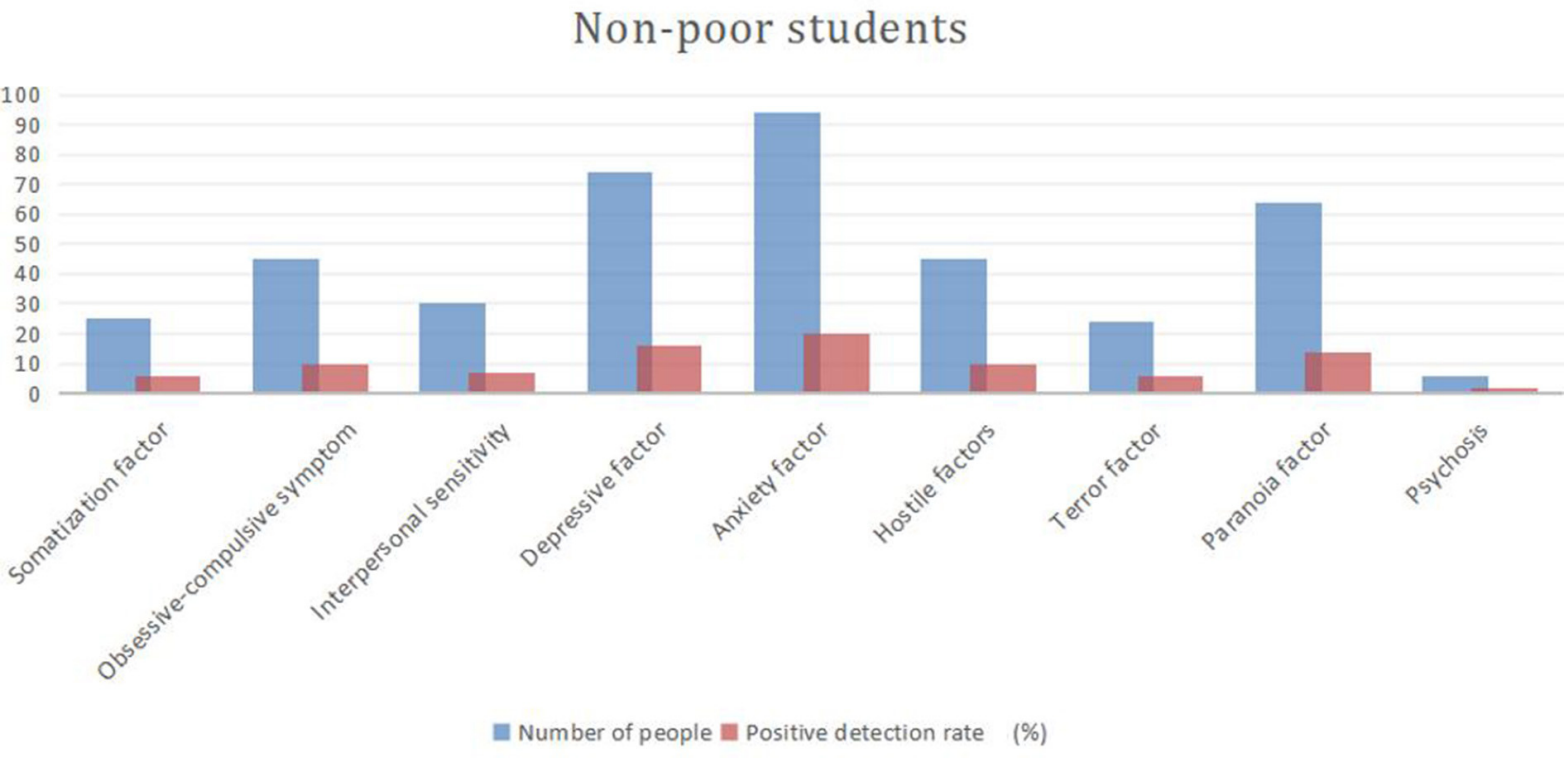
Positive detection rates of SCL-90 factors among non-poor students.

On the SCL-90 scale, a factor higher than two is positive, indicating that the subject is “symptomatic,” and the percentage is called the positive detection rate. Figures 2, 3 and Table 3 show that the positive detection rate in needy students is higher than in non-poor students, for depression, anxiety, interpersonal sensitivity, paranoia, compulsion, symptoms, hostility, somatization, terror, and psychosis.

**Table 3:** Positive detection rates of SCL-90 factors among poor and non-poor students.

**Factor**	**Needy students (78)**		**Non-poor students (452)**	
	**Number of students**	**Positive detection rate (%)**	**Number of students**	**Positive detection rate (%)**
Somatization factor	4	9	25	6
Obsessive-compulsive symptom	9	18	45	10
Interpersonal sensitivity	13	22	30	7
Depressive factor	18	30	74	16
Anxiety factor	15	28	94	20
Hostile factors	3	13	45	10
Terror factor	5	8	24	6
Paranoia factor	10	20	64	14
Psychosis	1	3	6	2

#### Distribution of detection rates of psychological problems

To assess the presence of a psychological condition, a rough and straightforward method is to check whether the factor score exceeds three points. Generally, a factor score of more than three points, or a total average score of greater than or equal to three points of positivity, indicates the possibility of a psychological condition of average or above; the higher the score, the lower the level of mental health. The results of this study indicated that a total of 48 students may have moderate or above-average psychological problems accounting for 9.1% of all students. From Table 4, it can be seen that there is no significant difference in the detection rate of psychological problems between male and female students. However, the detection rate of psychological problems in needy students is significantly higher than that of non-poor students by 6.1%. There is a specific difference between urban and rural students, which may be why most poor students come from rural areas.

**Table 4:** Distribution of the detection rate of psychological problems.

	**Male**	**Female**	**Urban**	**Rural**	**Poverty**	**Non- poverty**
Number of students	25	23	25	23	12	36
Proportion (%)	7.9	8.7	7.4	9.6	13.4	7.3

### Survey results and analysis of the social support rating scale

The results showed no significant difference between the objective support scores of the needy students in higher vocational colleges and the non-poor students. In contrast, the emotional support and the utilization of support scores were significantly lower in students from low income backgrounds than those in non-poor students. It shows that poor students in higher vocational colleges are not satisfied with the level of respect and support they receive and are not good at controlling and using social support to solve problems. Compared with non-poor college students, poor students lack awareness of using social support and do not tend to develop habits of using various support sources, as seen in Table 5.

**Table 5:** Comparison of the social support dimensions between needy students and non-poor students.

	**Needy students**	**Non-poor students**	***t***
	***n* = 78**	***n* = 452**	
Objective support	10.23 ± 2.45	10.18 ±	0.26
Subjective support	20.37 ± 3.11	20.78 ± 3.23	−1.17
Support utilization	7.56 ± 1.84	7.85 ± 1.77	−1.19

### Analysis of the social support dimension of needy college students and the various factors of SCL-90

The Pearson's correlation analysis between the social support dimension and the SCL-90 factors was between −0.311 and 0.135, indicating that the former had a significant negative relationship with the latter, that is, the higher the score of objective support, emotional support, and support utilization among the needy students, the lower the score of the SCL-90 factors, as seen in Table 6.

**Table 6:** Analysis of social support dimension and SCL-90 factors.

**Factor**	**Objective support**	**Subjective support**	**Support utilization**
Somatization factor	−0.164*	−0.221*	−0.181**
Obsessive-compulsive symptom	−0.122**	−0.273**	−0.192**
Interpersonal sensitivity	−0.189**	−0.301**	−0.233**
Depressive factor	−0.211**	−0.298**	−0.274**
Anxiety factor	−0.158**	−0.277**	−0.201**
Hostile factors	−0.168**	−0.245*	−0.219**
Terror factor	−0.091*	−0.189**	−0.171**
Paranoia factor	−0.152**	−0.199**	−0.164**
Psychosis	−0.170*	−0.278**	−0.228**

*Indicates *P* < 0.05, **Indicates *P* < 0.01.

## Analysis and conclusion

### Comparison of the mental health status of needy university students

Through comparative analysis of two needy students and non-poor students from two universities in Hainan city, we found that the SCL-90 factors were significantly different between the two groups. Except for the terror factor where the differences were not significant, in the remaining eight factors, the average scores of the needy students were significantly higher. The students from low income backgrounds also had a higher positive detection rate of psychological problems indicating that the mental health of students from poor backgrounds was relatively low.

There are many reasons for mental health problems, but the results from this study show that poverty is a significant factor. Furthermore, in this study there were also a number of “double needy students” who experience “psychological poverty” on university campuses, and this problem cannot be ignored. Needy students often worry about life and the future and are prone to inferiority, anxiety, depression, paranoia, pessimism, poor interpersonal communication, and other psychological problems.

Due to their financial status, the inferiority complex in some needy students becomes acute. These students are afraid of other students looking down on them, worried about exposing their weaknesses, unwilling to let others know their plight, are introverted, unsociable, and reluctant to actively communicate with classmates and teachers, hesitant to participate in collective activities, have fewer friends, prefer to be alone, get into sensitive interpersonal relationships rather easily, and suffer from depression and other mental health problems. Because of the heavy financial burden on the family, needy students often worry about living expenses, tuition fees, family members, the family's economic situation, and their academic future. Due to the psychological impact of living long-term under impoverished conditions, suffering from poverty, enduring the enormous social gap between the rich and the needy, and facing contempt because of their background, poor students often find it difficult to adjust, develop an aversion toward society and the rich, and in some cases end up with feelings ranging from hostility to revenge and paranoia. Economic poverty, prolonged inferiority, anxiety, depression, and paranoia are likely to affect physical health, and combined with poor diet and malnutrition, directly affect these students' physical and mental health. In addition, only a small number of needy students have the opportunity to work, while others try for scholarships, or work and struggle to find time to study, exhausting themselves physically and mentally.

### Comparison of social support between needy students and non-poor students

By comparing the various dimensions of social support between needy students and non-poor students, this study found that the objective support scores between both sets of students were comparable. In contrast, the score for supervisor support and utilization of support was significantly lower in needy students than in non-poor peers.

Human beings are an embodiment of their social relations. As social animals, human beings need to communicate with members of society and obtain material and emotional support from social members and organizations. Research by psychological professionals shows that social support is an essential way of solving psychological conflict and crises; because there exist interactive and mutually influential ties between people, they must get support and help from each other to solve the crises or difficulties they face.

The concept of social support mentioned in this paper can be divided into objective (material) support and emotional support. Different scholars have different views on the importance of the two kinds of support, but most scholars argue that emotional support is more meaningful than material support. Although emotional support is not tangible in reality, “perceived reality is a psychological reality, and it is a psychological reality, as the actual intermediary variable, that affects people's behavior and development.” However, this does not mean that material support is meaningless. Although there are significant individual differences in the social support of subjective experience, it always has a specific objective basis. The government also has a corresponding system of “award, loan, assistance, supplement and reduction” and other funds for needy students. However, in this study, there were no significant differences between the needy and non-poor students in their objective support scores. This paper believes that perhaps the funding is minimal, or the scholarship amount is too little, such that it makes no difference to needy students. Furthermore, the students could perceive it as a “hand-out” which damages their self-esteem and makes them feel that society does not care about them.

In this study, students from low income backgrounds experienced significantly lower levels of emotional support than non-poor students, and their use of social support was also significantly lower. They are also not good at using social support to solve problems, compared with non-poor students, as they lack the use of social support consciousness and the habit of accessing support sources. This could be related to their family background and their cognition and evaluation deviation, stemming from inferiority, depression, and interpersonal sensitivity, which stops them from using social support. To address the mental health issue in poor students, it is therefore important to strengthen and increase funding, while simultaneously ensuring respect, support, and understanding of the situations that poor students struggle with.

### Analysis of the social support dimension of poor college students and the various factors of SCL-90

The correlation analysis of the social support dimension of poor students and the SCL-90 factors showed a significant negative relationship between both. This implies that if more help or support is obtained, it is easier for students to deal with their crises or difficulties, which in turn aids the development of their attitudes in a benign and healthy direction.

The relationship between social support and health has been studied extensively in academia, and most scholars believe that good social support is good for health. In contrast, the existence of poor social relations is harmful to physical and mental health. On the one hand, social support serves as a buffer and protects individuals during stressful times. On the other, it is essential for maintaining their overall emotional countenance. The research in this paper is consistent with this conclusion.

In essence, successful social support should not only be one-dimensional support from the subject to the object but should be a process of two-sided interaction or a process of “mutual construction” between supporters and those who receive that support; that is, vulnerable groups are no longer passive recipients of social support, and after internalizing the social support they have received, they must respond in kind and actively invest social support systems that are conducive to their development. Inter-constructed social support emphasizes the conscious and active construction of the vulnerable groups, which is the practical process of the social support of the vulnerable groups from spontaneous to conscious, and from passive to active. Therefore, while society provides social support to needy students, it should also encourage poor students to seek and accept social support and assistance with a positive mindset. This will address students' loneliness and helplessness, and help them overcome their difficulties within a short period of time. It is also beneficial for their mental health as avoiding or rejecting care and help from others can equally affect physical and mental health.

## Research conclusions

The conclusions of this paper are as follows:

1. The SCL-90 symptom test reveals that the mental health of needy students in colleges and universities is relatively low. There are different degrees of psychological problems, and needy students are prone to obsessive-compulsive disorder, interpersonal sensitivity, depression, anxiety, hostility, paranoia, psychosis, and other psychological problems that also affect physical health.
2. The comparison of the utilization of various types of social support between needy and non-poor students shows that needy students are not satisfied with the levels of respect, support, and understanding they experience. They are not good at being in control and using social support to solve problems.
3. The correlation between social support and SCL-90 factors of needy students in colleges and universities indicates a significant negative correlation between the two, demonstrating that good social support is of immense significance for maintaining a generally good emotional experience. Therefore, to effectively ensure the mental health of needy students, it is necessary to establish an adequate social support system and encourage poor students to make full use of social support.

Schools should realize that the social personalities and psychological development of adolescents are malleable. Support for boosting the mental health of students from low income backgrounds should by no means be limited to psychological counseling. Higher educational institutes should provide a comprehensive educational environment including shaping campus culture, providing ideological and moral education, and establishing a financial assistance system.

### Practical and policy implications

1. Improve the existing student assistance system: Economic stress is at the root of the psychological problems of needy students. “To cure the disease, cure the root cause.” Therefore, providing financial assistance to needy students to relieve their worries is the best way to solve their psychological problems. At present, the support measures commonly offered by colleges and universities in China are: first, the establishment of “awards, loans, assistance, supplements, reductions” and other capital assistance initiatives. Second, the establishment of a “green channel” to facilitate prospective students from economically weaker backgrounds to go through enrollment procedures by ensuring that no new student is unable to enroll due to economic difficulties.

Due to limited benefits and low subsidy amounts, needy students often do not seek additional support measures. To address the financial problems needy students face, colleges and universities should make full use of the government's current policy of promoting student loans for college students. They should also set up special incentives and support funds for needy students through funding from relevant departments, actively seek public support and foreign donations to reduce tuition and miscellaneous fees, and offer living subsidies. Higher educational institutes must offer the most basic economic guarantee for needy students to complete their studies and alleviate the direct psychological pressure they are under due to economic difficulties. Universities can also advocate for non-governmental entities such as business houses, enterprises, and social groups to proactively establish a “counterpart support” relationship with poor students, which can ensure the smooth completion of studies for some needy students and cultivate specific skills required in enterprises aimed at alleviating their employment pressure to a certain extent.

The Hainan University School of Technical Supervision has a small number of needy students who receive financial support from enterprises; the enterprises regularly provide needy students with economic assistance and recruit students after graduation, who then work with them for a certain number of years. This study found that among students receiving assistance, there was no economic pressure and the students were optimistic, positive, and filled with gratitude that they would be accepted after their graduation to work in the enterprise that had funded their studies.

2. Strengthen the capacity of mental health work teams. Colleges and universities should train teachers engaged in mental health for college students. Through continuous training, they will gain theoretical insights, improve professional knowledge, and learn skills necessary for them to impart effective mental health education. It is also necessary to focus on relevant training of class teachers, counselors, and mentors on mental health content.

The mental health education workforce in some colleges and universities in China is particularly weak, and quite often mental health education is offered within ideological and political education resulting in needy students already beset with mental health problems being further confused between ideological problems and life problems without timely and correct guidance. Although ideological and political education has the potential to address some issues relating to students' mental health, there is a big difference between the two. Ideological and political education often focuses on the ideological level and strives to improve students' ideological awareness and moral character. The mental health bias on the subconscious helps to exert a role in the whole process of the students' cognition and emotion. It guides them to maintain a healthy psychological state and develop good psychological conditions for receiving correct ideological education, thereby changing students' mental outlooks.

3. Attach importance to the mental health education of needy students. The school environment and education play a pivotal role in the formation of the student's personality. Needy students tend to mature physically and mentally in the middle of their adolescence, but they have not yet formed a completely healthy personality. Most of the current challenges of the prevalent mental health of students are caused by personality disorders. At the university level, most students only encounter common psychological problems. The popular Chinese saying cautions “mend the pen after the sheep is dead,” calling for timely and preventive action, and as this saying observes, mental health education should commence while students are still at school to prevent common psychological problems at the university level.

A facility such as a Student Psychological Counseling Room should be a permanent feature in an institution catering to the mental health needs of college students. Counseling staff should include teachers with specialist knowledge, rich practical experience, and genuine concern for the mental wellbeing of all students. The counseling room should invest in the daily mental health education of students, help in establishing students' mental health census and health files, undertake individual and group psychological counseling, schedule counseling tasks for students, and fully care for and support students by building an environment conducive to students' self-growth.

## Data availability statement

The raw data supporting the conclusions of this article will be made available by the authors, without undue reservation.

## Ethics statement

Ethical review and approval was not required for the study on human participants in accordance with the local legislation and institutional requirements. Written informed consent from the participants or participants legal guardian/next of kin was not required to participate in this study in accordance with the national legislation and the institutional requirements.

## Author contributions

The author confirms being the sole contributor of this work and has approved it for publication.

## Conflict of interest

The author declares that the research was conducted in the absence of any commercial or financial relationships that could be construed as a potential conflict of interest.

## Publisher's note

All claims expressed in this article are solely those of the authors and do not necessarily represent those of their affiliated organizations, or those of the publisher, the editors and the reviewers. Any product that may be evaluated in this article, or claim that may be made by its manufacturer, is not guaranteed or endorsed by the publisher.

## References

[B1] Battagliese G. Caccetta M. Luppino O. I. Baglioni C. Cardi V. Mancini F.. (2015). Cognitive-behavioral therapy for externalizing disorders: a meta-analysis of treatment effectiveness. Behav. Res. Ther. 75, 60–71. 10.1016/j.brat.2015.10.008 26575979

[B2] Carsley D. Khoury B. Heath N. L. (2018). Effectiveness of mindfulness interventions for mental health in schools: a comprehensive meta-analysis. Mindfulness 9, 693–707. 10.1007/s12671-017-0839-2

[B3] Chiang K. J. Tsai J. C. Liu D. Lin C. H. Chiu H. L. Chou K. R.. (2017). Efficacy of cognitive-behavioral therapy in patients with bipolar disorder: a meta-analysis of randomized controlled trials. PLoS ONE 12, e0176849. 10.1371/journal.pone.0176849 28472082 PMC5417606

[B4] Chibanda D. Mesu P. Kajawu L. Cowan F. Araya R. Abas M. A.. (2011). Problem-solving therapy for depression and common mental disorders in Zimbabwe: piloting a task-shifting primary mental health care intervention in a population with a high prevalence of people living with HIV. BMC Public Health 11, 1–10. 10.1186/1471-2458-11-828 22029430 PMC3210104

[B5] Cohen J. A. Deblinger E. Mannarino A. P. (2018). Trauma-focused cognitive behavioral therapy for children and families. Psychother. Res. 28, 47–57. 10.1080/10503307.2016.1208375 27449400

[B6] Cristea I. A. Gentili C. Cotet C. D. Palomba D. Barbui C. Cuijpers P.. (2017). Efficacy of psychotherapies for borderline personality disorder: a systematic review and meta-analysis. JAMA Psychiatry 74, 319–328. 10.1001/jamapsychiatry.2016.4287 28249086

[B7] Ebert D. D. Zarski A. C. Christensen H. Stikkelbroek Y. Cuijpers P. Berking M.. (2015). Internet and computer-based cognitive behavioral therapy for anxiety and depression in youth: a meta-analysis of randomized controlled outcome trials. PLoS ONE 10, e0119895. 10.1371/journal.pone.0119895 25786025 PMC4364968

[B8] Farahmand F. K. Grant K. E. Polo A. J. Duffy S. N. DuBois D. L. (2011). School-based mental health and behavioral programs for low-income, urban youth: a systematic and meta-analytic review. Clin. Psychol.: Sci. Pract. 18, 372. 10.1111/j.1468-2850.2011.01265.x

[B9] Gonzalez-Suarez C. Worley A. Grimmer-Somers K. Dones V. (2009). School-based interventions on childhood obesity: a meta-analysis. Am. J. Prev. Med. 37, 418–427. 10.1016/j.amepre.2009.07.012 19840696

[B10] Grant J. S. Elliott T. R. Giger J. N. Bartolucci A. A. (2001). Social problem-solving abilities, social support, and adjustment among family caregivers of individuals with a stroke. Rehabil. Psychol. 46, 44. 10.1037/0090-5550.46.1.44

[B11] Gronholm P. C. Nye E. Michelson D. (2018). Stigma related to targeted school-based mental health interventions: a systematic review of qualitative evidence. J. Affect. Disord. 240, 17–26. 30041074 10.1016/j.jad.2018.07.023

[B12] Hofmann S. G. Asnaani A. Vonk I. J. Sawyer A. T. Fang A. (2012). The efficacy of cognitive behavioral therapy: a review of meta-analyses. Cognit. Ther. Res. 36, 427–440. 10.1007/s10608-012-9476-1 23459093 PMC3584580

[B13] Linardon J. Wade T. D. De la Piedad Garcia X. Brennan L. (2017). The efficacy of cognitive-behavioral therapy for eating disorders: a systematic review and meta-analysis. J. Consult. Clin. Psychol. 85, 1080. 10.1037/ccp0000245 29083223

[B14] Miller W. R. Rollnick S. (2012). Motivational Interviewing: Helping People Change. Guilford Press.

[B15] Miller W. R. Rose G. S. (2015). Motivational interviewing and decisional balance: contrasting responses to client ambivalence. Behav. Cogn. Psychother. 43, 129–141. 10.1017/S1352465813000878 24229732

[B16] Nezu A. M. D'Zurilla T. J. (2006). Problem-Solving Therapy: A Positive Approach to Clinical Intervention. Springer Publishing Company.

[B17] Nezu A. M. Nezu C. M. D'Zurilla T. (2012). Problem-Solving Therapy: A Treatment Manua. Springer Publishing Company.

[B18] Riper H. Andersson G. Hunter S. B. Wit d. e. Berking J. Cuijpers M.. (2014). of comorbid alcohol use disorders and depression with cognitive-behavioral therapy and motivational interviewing: a meta-analysis. Addiction 109, 394–406. 10.1111/add.12441 24304463 PMC4227588

[B19] Ruocco S. Gordon J. McLean L. A. (2016). Effectiveness of a school-based early intervention CBT group programme for children with anxiety aged 5–7 years. Adv. School Mental Health Promot. 9, 29–49. 10.1080/1754730X.2015.1110495

[B20] Sanchez A. L. Cornacchio D. Poznanski B. Golik A. M. Chou T. Comer J. S.. (2018). The effectiveness of school-based mental health services for elementary-aged children: a meta-analysis. J. Am. Acad. Child Adolesc. Psychiatry. 57, 153–165. 10.1016/j.jaac.2017.11.022 29496124

[B21] Trauer J. M. Qian M. Y. Doyle J. S. Rajaratnam S. M. Cunnington D. (2015). Cognitive behavioral therapy for chronic insomnia: a systematic review and meta-analysis. Ann. Intern. Med. 163, 191–204. 10.7326/M14-2841 26054060

[B22] Wade S. L. Kurowski B. G. Kirkwood M. W. Zhang N. Cassedy A. Brown T. M.. (2015). Online problem-solving therapy after traumatic brain injury: a randomized controlled trial. Pediatrics 135, e487–e495. 10.1542/peds.2014-1386 25583911 PMC4306792

